# A Retrospective Observational Study Examining the Effect of Thoracic Epidural and Patient Controlled Analgesia on Short-term Outcomes in Blunt Thoracic Trauma Injuries

**DOI:** 10.1097/MD.0000000000002374

**Published:** 2016-01-15

**Authors:** Edward James Baker, Geraldine Ann Lee

**Affiliations:** From the Emergency Department, Kings College Hospital, London, UK (EJB); Florence Nightingale Faculty of Nursing & Midwifery, Kings College London, London, UK (EJB); and Florence Nightingale Faculty of Nursing & Midwifery, Kings College London, London, UK (GAL).

## Abstract

Effective analgesia in the early stages after any major traumatic event remains pivotal to optimal trauma management. For patients with significant thoracic injuries, this is paramount to ensure ongoing efficient respiratory function. The aim of this study was to investigate the use of analgesic modes in the management of patients with a primary thoracic injury and blunt mechanism of injury. By understanding variables that influence the use of varying analgesic modes and influence the development of pulmonary complications, there should be more uniform evidence-based prescription in the future.

This retrospective study considered analgesic use in patients admitted after blunt thoracic injuries at one major trauma center over a 2-year period. Pulmonary complications measured included both infective and ventilator-associated failure. Univariate and multivariate analyses were used to identify patient and injury severity characteristics and their association with respiratory complications.

A total of 401 cases were reviewed and analyzed: 159 received Patient Controlled Analgesia (PCA), 32 received PCA and epidural analgesia (EA), 6 received EA alone, and 204 received interval-administered analgesia. There were no significant differences in the rates of complication when compared between analgesic modes. Patients who developed pneumonia had significantly increased number of thoracic fractures and underlying organ injury (*P* < 0.05). Logistic regression analysis highlighted duration of intercostal drain insertion (OR 1.377, *P* = 0.001) and premorbid cardiac disease (OR 2.624, *P* = 0.042) and ICU length of stay (OR: 1.146, *P* < 0.001) as significant predictors of developing pneumonia in this patient group.

Examining the different analgesic modes, this study failed to identify a particular analgesic mode that was more effective in preventing pulmonary complications in blunt thoracic injuries. However, variables that may influence usage of different analgesic modes and high-risk groups for the development of pneumonia were identified. Further work is warranted to consider the long-term benefits of analgesia in patients post-blunt thoracic injuries.

## INTRODUCTION

Trauma remains the leading cause of death for both men and women under the age of 44 years in high-income countries and is a unique process in that it continues to have increasing morbidity and mortality rates with significant financial cost.^1–3^ It is also a major contributor to death and disability in low- and middle-income countries.^4,5^ Blunt chest trauma is responsible for >15% of all trauma admissions in the UK with mortality rates of 4% to 20%.^6,7^ Characterized by injury that does not involve opening of the chest wall, blunt chest trauma can vary in severity from minor hematoma or isolated single rib fractures, to severe crush injuries compromising the thoracic structure and leading to potentially fatal respiratory failure. Therefore due to its very nature, blunt trauma injuries are associated with significant mortality and morbidity.

Bony injury to the thoracic cavity is a significant indicator of underlying organ injury,^8,9^ and a significant link between injury severity and age has been observed that increases with each additional rib fracture.^10–13^ Management of the most severe injuries routinely requires invasive positive pressure ventilation, as the mechanisms of normal negative pressure ventilation will be ineffectual.^14–16^ Difficulty occurs with these injuries when they are present after significant chest wall injury with normal respiratory function because respiratory compromise often developing 48 to 92 h after admission resulting in respiratory failure, pneumonia, and pleural sepsis.^17,18^

From a management perspective, late onset of respiratory complications along with premorbid state and severity of illness adds to the complexity of trauma care.^17^ One of the cornerstones of trauma care is pain management^19,20^ and previous studies have demonstrated improved clinical outcomes and self-assessed pain scores when either patient controlled analgesia (PCA) or epidural analgesia (EA) is utilized after significant chest trauma.^20–23^ A meta-analysis of 8 randomized control trials (n = 232) demonstrated a significant reduction in ventilation requirements in patient managed with EA when compared to other forms of analgesia.^24^ In the United States, a large national cohort study of blunt thoracic injuries saw reduced adjusted mortality at 30 days (OR 0.08; 95% CI 0.01–0.43) 90 days (OR 0.09; 95% CI 0.02–0.42) post injury in those managed with EA.^25^ A further randomized trial in traumatic flail chest patients also demonstrated a reduction in ICU length of stay and a reduced need for non-invasive ventilation in the intervention group using rib fixation but due to patients being sedated, it was not possible to measure pain.^26^

The aim of this study was to investigate the use of analgesic modes in the management of patients with a primary thoracic injury and blunt mechanism. The analgesic modes initially considered for analysis included PCA and EA used in isolation based on the treating clinician's decision. These variables were assessed comparably against age (≥60 years old) and the presence of nosocomial infective respiratory complications (pneumonia) as the literature has identified these variables as having high predictive values for increased mortality and morbidity.^10,27–29^ These data were used to examine the effect of analgesic mode on the development of respiratory complications and help identify relevant variables that may predict respiratory complications in this patient group. It was hypothesized that effective analgesia would reduce the onset of respiratory complications in patients with blunt thoracic injuries.

## METHODS

A retrospective review of patients with blunt chest trauma admitted to one major trauma centre (MTC) in southeast London (UK) between January 2012 and March 2014 was undertaken. King's College Hospital is a large tertiary referral centre with a level 1 trauma centre and received 1856 major trauma patients in 2013 increasing to 1986 patients in 2014. The service expanded in 2013 to include the Southeastern counties of the UK. The Trauma Audit and Research Network identified 515 patients who had been coded with primary blunt thoracic injuries.

The inclusion criteria were individuals aged 16 years or older, presence of 1 or more thoracic fractures (including ribs, sternum, scapular, and clavicular fractures) and admitted for >24 h. Patients who died within 24 h of admission to hospital, those under the age of 16 years, and those with penetrating injuries were excluded. Patients with injury severity score (ISS) <15 were also included in the data collection in this study. A technique of convenience sampling was used in this observation study.

For the purpose of this study the data collected looked at patients receiving morphine-based PCA set to deliver 1 mg with a 10 min lock out and no continuous infusion. PCA's that varied from this prescription where excluded from this data collection. Epidurals included were fentanyl and bupivicaine based that run as a continuous infusion. It is not routine practice at this trauma center to undertake parvertibral blocks or run patient controlled epidural analgesia.

Data were collected from the hospital's electronic patient record system using a pre-designed data collection tool based on the previous literature.^20,21^ This included demographic data (age, gender, relevant co-morbidities), predictors of trauma severity (ISS, number of thoracic fractures, location of fractures, including a number of fractures on 1 bone, underlying organ injuries, presence of prehospital thoracostomy, and inter-costal drain placement).^30^ Primary outcomes measured included respiratory complications that included pneumonia, type 1 and type 2 respiratory failure, pulmonary embolism, and lower respiratory tract symptoms and secondary outcomes measured included hospital and intensive care length of stay and 30 day mortality postdischarge.^31^ Missing data were excluded from study analysis.

Data were entered onto a database and statistical analysis was undertaken using SPSS v21. Statistical data were presented as descriptive and inferential statistical data and presented according to the Strengthening the Reporting of Observational Studies in Epidemiology statement.^32^ Demographic information was presented as percentages and number of cases and means with standard deviations were applied for parametric continuous data. The statistical tests applied were Student's *t* test for parametric data, and Mann–Whitney and chi-squared analysis for binary nonparametric data. ANOVA was used for multivariate continuous data and chi-squared analysis for multivariate binary data. Logistic regression analysis was undertaken to investigate the potential contribution of relevant confounding variables on the development of respiratory complications. Linear regression analysis was undertaken to identify variables that indicated a prolonged acute hospital length of stay. A *P* value of <0.05 was deemed statistically significant. Statistical support was provided through the Med-stats team at King's College Hospital/Kings College London (UK).

Institutional approval to undertake the study was obtained from King's College Hospital and King's College London, before the commencement of data collection. For the purpose of this study interval administered analgesia included oral, intramuscular, and subcutaneous and narcotic agents given intermittently or Pro Ra Nata.

## RESULTS

**FIGURE 1:**
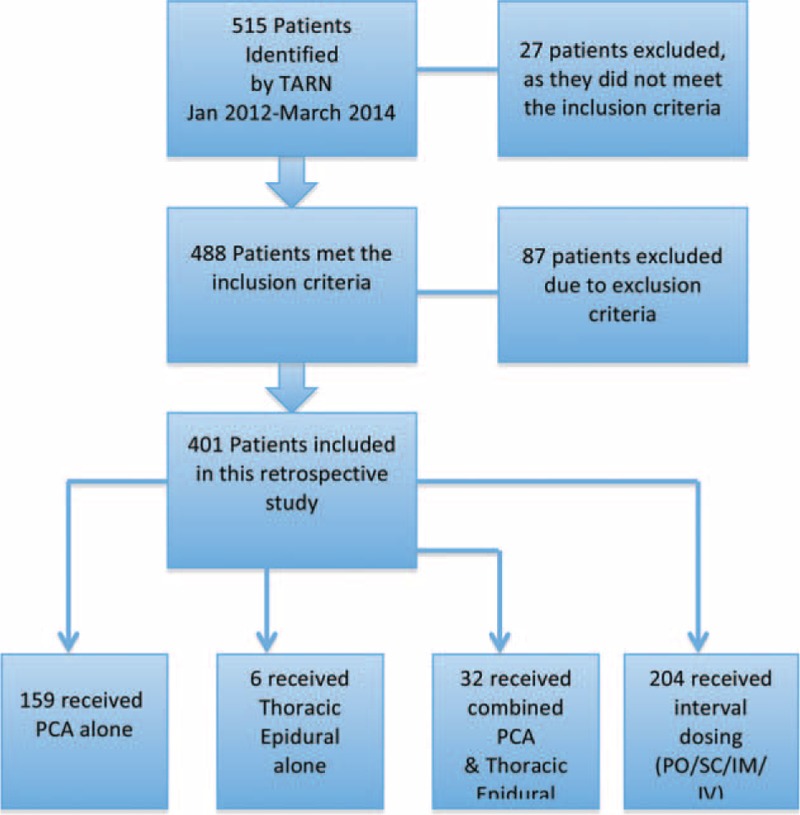
Flowchart of patient selection.

A total of 488 patients were identified as meeting the inclusion criteria and of these 87 were excluded as they were under the age of 16 years, died within 24 h of admission or had penetrating injuries to the thoracic cavity. Of the remaining 401 patients, 159 received PCA alone, 6 patients received EA, 32 received a combined analgesic of EA and PCA and 204 patients received interval-administered analgesics (Figure 1).

The demographic data for 401 patients admitted to King's College Hospital after significant blunt chest trauma is presented in Table 1. The mean age of patients included was 48.9 (±19.2) years, majority were men (77%) and the mean ISS was 25.3 (±11.9). The mean number of thoracic fractures was 6.6 (± 5.4) and the average total length of hospital stay was 17.6 days (± 22.6). The mortality was 7% (n = 28).

**TABLE 1:**
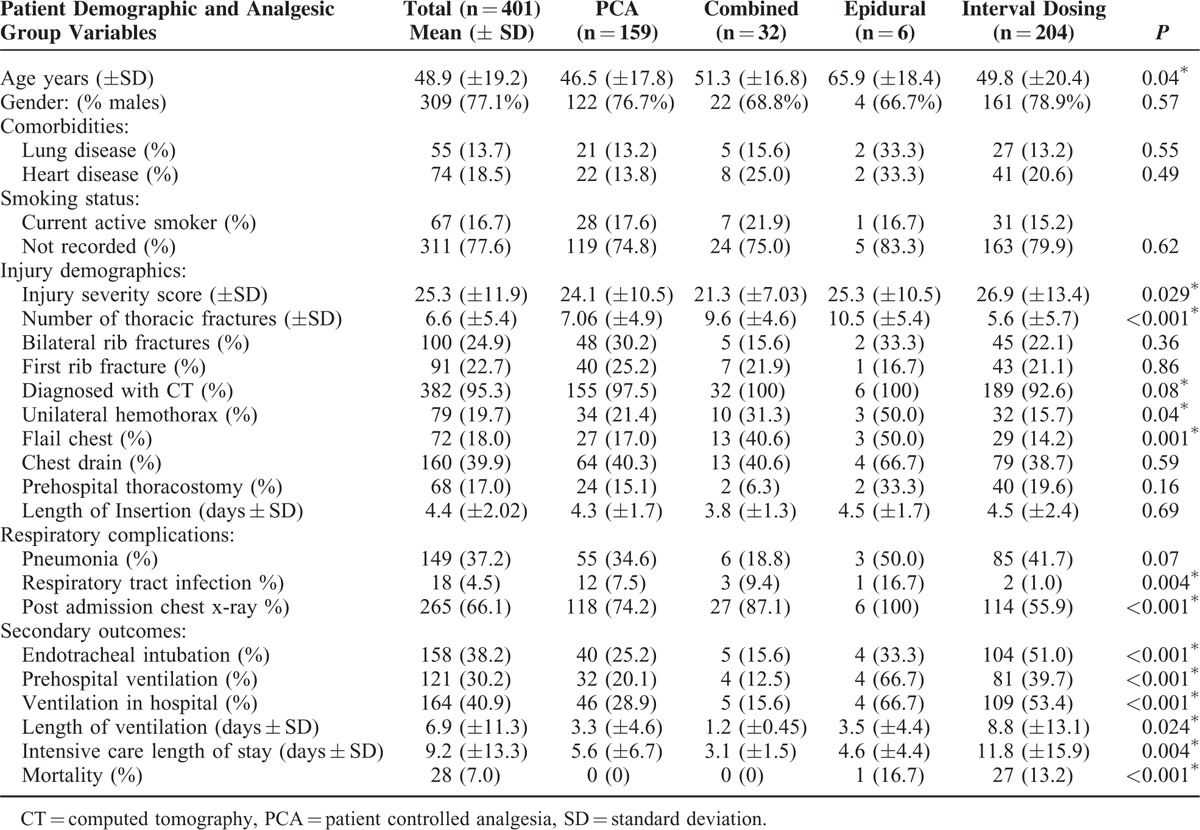
Demographic and Analgesic Group-Specific Data

ISS were significantly higher in those patients managed with EA alone and interval analgesia when compared to those who received PCA alone and a combine PCA and thoracic epidural (25.3 [±10.5] and 26.9 [±13.4] vs 24.1 [± 10.5] and 21.3 [±7.03], *P* = 0.029). Likewise, those patients managed with combined PCA and thoracic epidural and EA alone had significantly higher numbers of thoracic fractures when compared to those who received PCA alone or interval administered analgesics (9.6 [±4.6] and 10.5 [±5.4] vs 7.06 [±4.9] and 5.6 [±5.7], *P* < 0.001). There were also significant variances in the distribution of flail segments when compared between PCA and EA (17.0% vs 50.0%, *P* = 0.001).

Patients who developed pneumonia after admission to hospital presented initially with more thoracic fractures on CT (8.1 [±6.1] vs 5.7 [±4.8], *P* < 0.001) and higher ISS when compared to those who did not develop pneumonia (29.1 [±12.0] vs 23.0 [±11.3], *P* < 0.001). These patients were also more likely to have bilateral rib fractures (32.2% vs 20.7%, *P* = 0.03) and unilateral lung contusions (38.9% vs 28.3%, *P* = 0.04). Chest drain placement, prehospital thoracostomy, and duration of ICD placement were also significantly increased in patients who developed post admission pneumonia (*P* = 0.002) (Table 2). When differences between patients ≥60 years and those <60 years, the presence of comorbid conditions were significantly more prevalent in those patients ≥60 years (lung disease: 24.0% vs 9.29%, *P* < 0.001). Patients also had less underlying organ injuries, with no difference in ISS (25.0 vs 25.5, *P* = 0.73) but had significantly higher rates of pneumonia (47.9% vs 2.5%, *P* = 0.005) and mortality (13.2% vs 4.29%, *P* = 0.002) when compared to younger patients <60 years.

**TABLE 2:**
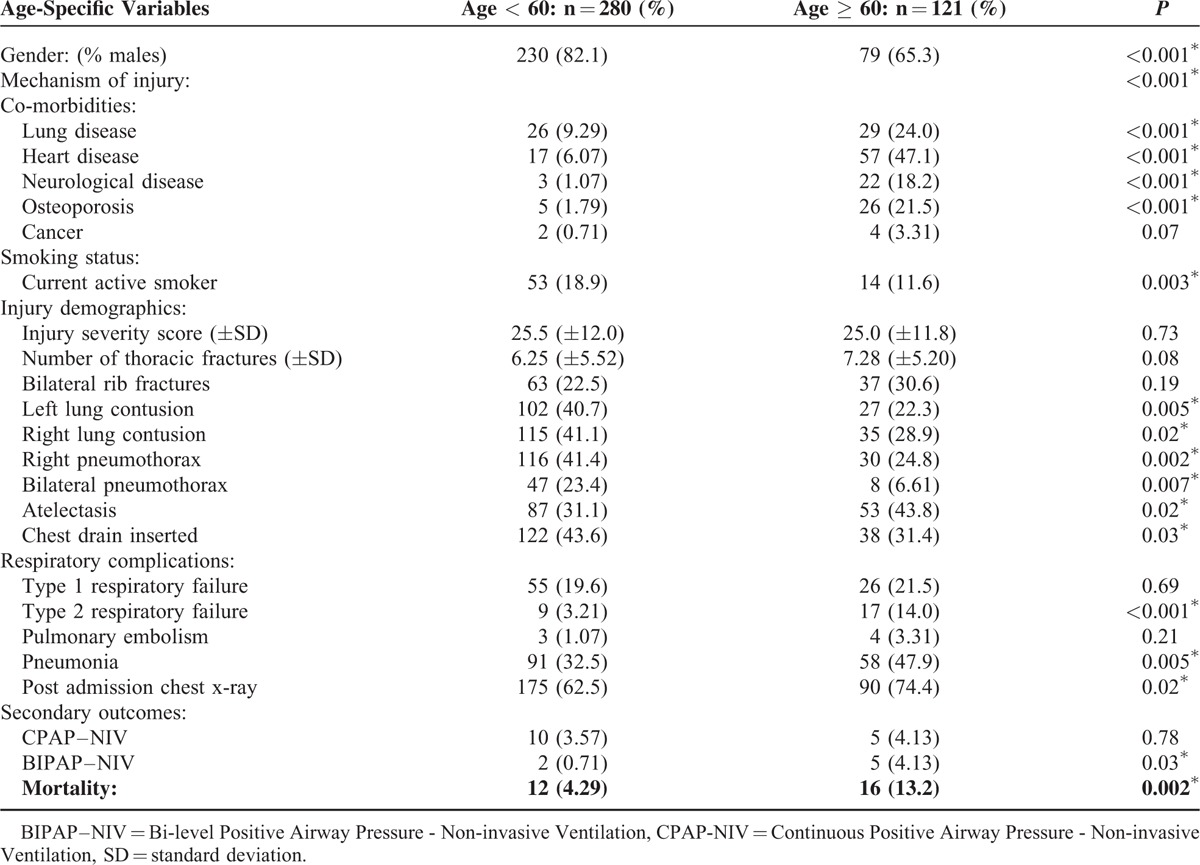
Age-Specific Variables for Those Patient ≥60

There was no statistically significant difference in the development of any respiratory complications when compared to the differing analgesic modes but there were significantly more episodes of type 1 and type 2 respiratory-failure recorded in patients diagnosed with pneumonia (Table 3). These patients also had significantly higher requirements for ventilator support including non-invasive, endo-tracheal ventilation, and prolonged ICU length of stay.

**TABLE 3:**
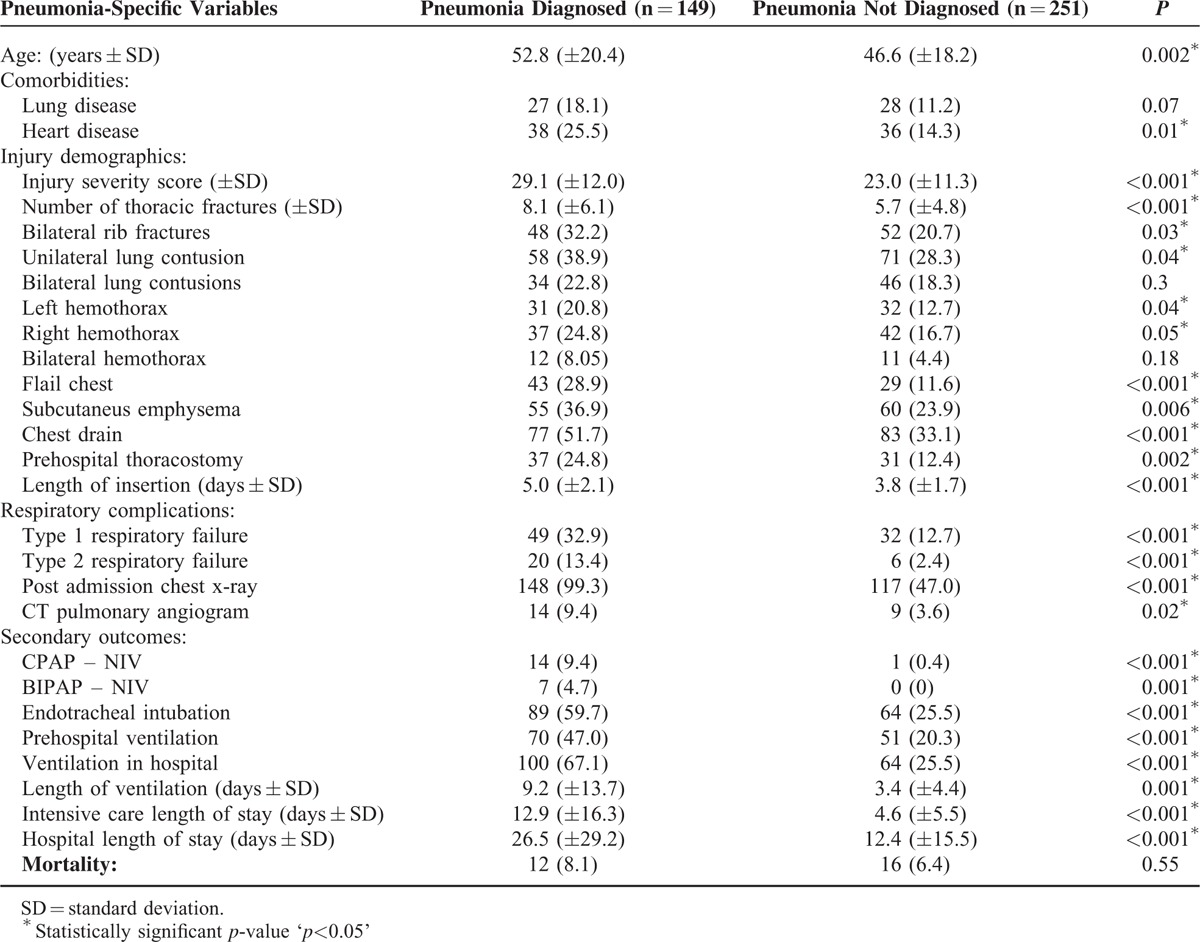
Pneumonia-specific Variables of Injury Severity and Outcomes

Logistic regression analysis was used to identify variables that predicted pneumonia (Table 4). Analysis identified that duration of ICD insertion (OR 1.377 [95%CI 1.142–1.66], *P* = 0.001); ICU length of stay (OR 1.146 [95%CI 1.08–1.216], *P* < 0.001); and pre-morbid cardiac disease (OR 2.624 [95%CI 1.034–6.665], *P* = 0.04) as significant predictor variables for developing nosocomial pneumonia. The regression model adjusted for confounding variables including use of PCA or EA, ISS, total number of thoracic fractures, other premorbid conditions, bilateral rib fractures, and flail segments and hospital length of stay.

**TABLE 4:**
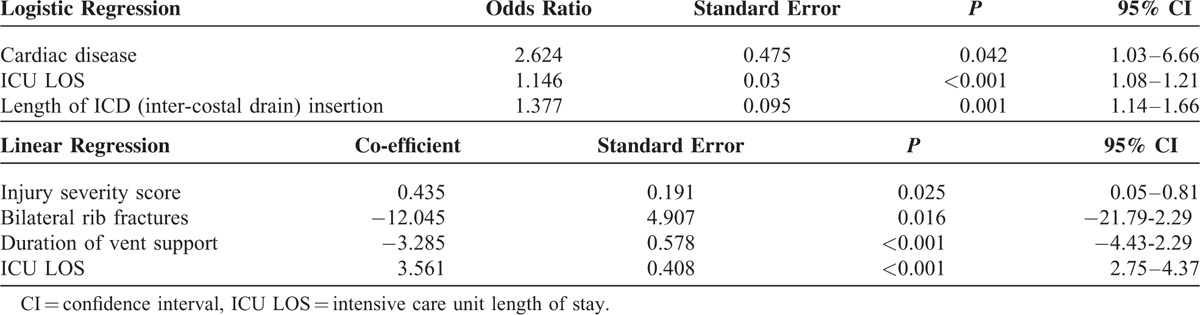
Logistic Regression Analysis for Predictors of Pneumonia and Linear Regression Analysis for Predictors of Increasing Hospital Stay

Similarly, linear regression analysis was used to identify variables that predicted length of hospital stay. Increasing ISS (coefficient: 0.435 [95%CI 0.055–0.815], *P* = 0.02), presence of bilateral rib fractures (coefficient: −12.045 [96%CI −21.791–−2.299], *P* = 0.01), increasing duration of required ventilation support (coefficient: −3.285 [95%CI −4.433–−2.299), *P* < 0.001), and increase intensive care length of stay (coefficient: 3.561 [95% CI 2.752–4.371], *P* = 0.001) all predicted a longer hospital length of stay.

## DISCUSSION

This retrospective study was able to demonstrate that blunt traumatic injury to the chest is commonly exacerbated by respiratory complications despite attempts to prevent this through effective analgesia. The demographic data collated highlighted the significant injuries this cohort sustains with the high number of thoracic fractures and underlying thoracic organ injury suggests that these patients had a high risk of developing post-admission complications.

Older patients were more likely to develop pneumonia and be managed with EA alone (*P* = 0.002 and *P* = 0.04 respectively) and age has previously been identified as a significant predictor of mortality and morbidity.^10,12,13,28,29,33–38^ Those >60 years had significantly higher rates of infective and ventilator-related respiratory complications and increased mortality (*P* < 0.05) but did not have increased critical care admissions contrary to Sirmali et al findings where improved morbidity and mortality was seen in patients with significant blunt thoracic injuries treated in a critical care environment.^38^ Blecher et al observed potential inconsistencies in the decision to admit that was heavily influenced by critical care capacity.^14^

Contrary to our initial hypothesis, we found that most patients managed with EA were also managed with PCA in a combined therapy. From the literature, it appears that investigation of the efficacy of combined EA and PCA at preventing respiratory complications has not previously been considered. This may be beneficial at reducing the opiate requirements of naive patients which can influence long-term addiction and opioid requirements.^39^

It was not possible to identify any association between rates of respiratory complications and the analgesic mode used in the early post injury phase. Previous retrospective studies have also failed to establish any significant association between analgesic mode and rates of infective and ventilation-related respiratory complications.^20,21^ Further robust research is required to understand the clinical benefits of EA and PCA in blunt chest trauma.^19^

In this study, only 9.5% of patients had EA successfully administered. It was not possible to access data relating to the patients who were referred for EA and did not undergo epidural insertion or the patients where epidural insertion failed. Previous studies have highlighted poor use of EA even in eligible patients. Wu et al and Todd et al highlighted uptake of EA in patients with blunt chest trauma at <20%.^40,41^ Yeh et al highlighted similar usage of this analgesic mode in their study (18%) and accounted for this as the literature is yet to define the most appropriate patient populations.^20^

Mortality in this study was recorded at 7% (n = 28) and when compared with pneumonia rates and analgesic mode mortality was not significantly higher in patients who developed pneumonia (*P* = 0.55). Mortality was significantly higher in the cohort who received interval-administered analgesia only (*P* < 0.001) and in those who were ≥60 years at time of injury (*P* = 0.002). Patients who received interval analgesia had higher ISS whereas those aged ≥60 years had lower ISS suggesting that the most severely injured patients were less likely to receive either EA or PCA. Equally, those aged ≥60 years had higher mortality despite lower injury severity, suggesting other variables are involved. Of the overall cohort, 77% were men predominantly of working age and this is in line with the literature. Trentzsch et al identified men to have higher incidence of complications including sepsis and multiorgan failure after traumatic injury.^42^ This may go some way to explain the rates of complications seen in this study but further work is required to fully understand the variable of gender in traumatic outcomes.

Pneumonia remains the most common cause of admission to a critical care environment.^18,43^ Those who survive intensive care treatment are at increased risk of developing long-term complications such as permanent kidney injury and dementia. This often will have a detrimental effect on patient's long-term functionality and quality of life.^44^ For this reason, quality of life assessment has become increasingly important as an outcome measure in research focusing on survival from critical injury and illness.^45–48^ Honselmann et al highlighted the high level of 1-year mortality for patients admitted to ICU with pneumonia.^49^ Likewise, Rainer et al highlighted a significantly decreased quality of life in major trauma patient's ≥65 years (OR 4.77) and those admitted to intensive care postinjury (OR 2.15).^50^ Further prospective work on the topic of respiratory complications post blunt chest trauma would benefit from consideration of long-term quality of life assessment and long-term pain perception.^51^

Patients who developed pneumonia after blunt chest injuries had significantly higher invasive ventilation requirements in hospital and in the prehospital environment (*P* < 0.001). These patients also had prolonged intensive care stays and required longer episodes of invasive and non-invasive ventilation (*P* < 0.05). Regression analysis confirmed intensive care length of stay as a significant predictor of developing pneumonia (OR 1.146 [95% CI 1.08–1.216], *P* < 0.001). Hua & Shah demonstrated reduced morbidity and mortality in patients who received early non-invasive ventilation, although this in itself is limited by the stringent contra-indications for using non-invasive ventilation.^16^

In this study, there were no significant difference in intercostal drain (ICD) requirements, prehospital thoracostomy, or ICD length of insertion when compared against each analgesic mode (*P* > 0.05). When compared against rates of pneumonia, those who developed pneumonia had significantly higher ICD requirements (*P* < 0.001), pre-hospital thoracostomy (*P* = 0.002), and longer length of insertion (*P* < 0.001). Previous literature has highlighted ICD placement as a significant predictor of respiratory complications (OR 2.791 [95% CI 1.205–6.462], *P* = 0.01) and increased intensive care admissions (PE 0.147; SE 0.060; *P* = 0.02) with prolonged hospital length of stay (*P* < 0.001).^14,20,52^

There were significantly higher ICD insertions in those <60 years compared to older patients (31.4% vs 43.6%, *P* = 0.03). This corresponds to the higher numbers of observed pneumo/haemothorax in the younger population. It is understandable that a prolonged ICD insertion increases the risk of infective complication and has been previously reported to increase the risk of complication in patients requiring ICD insertion in the ED from blunt chest injuries (OR 2.57 [95% CI: 1.27–5.21]).^53^ Regression analysis in this study highlighted the duration of ICD insertion as a significant predictor of pneumonia (OR 1.377 [95% CI 1.142–1.66] *P* = 0.001). Further research is required to understand the optimum point of ICD removal in blunt trauma patients, particularly in those where prehospital thoracostomy has been undertaken in a nonaseptic environment.

Premorbid heart disease was also identified as a significant predictor of pneumonia (OR 2.624 [95% CI 1.034–6.665] *P* = 0.04). This result is in line with Battle et al (2013) findings (OR 2.4 [95% CI 1.3–4.5], *P* = 0.007). Significant links have been identified between pneumonia and the subsequent development of cardiac disease in hospitalized patients.^54^ It remains unclear why heart disease may increase risk of developing pneumonia and further work is required to consider the effect of premorbid conditions in trauma outcomes.^12^ Linear regression analysis also highlighted variables that significantly increased hospital length of stay and these variables were all directly linked the injury severity.

This study examined the practice at 1 major trauma centre alone; this potentially means that the results might not be generalizable to other centers in the UK and internationally. The study population on reflection, is a rather heterogeneous group and this has made it difficult to ascertain whether either form of analgesia is more effective at preventing complications. This study also identified very small numbers receiving EA and this was limiting in the data analysis process.

Using a retrospective methodology for this study relied heavily on the accuracy of patient specific documentation and subjects the data to potential measurement and information bias. This study highlighted a number of areas of documentation practice where measurement bias could have been introduced (ie non-documentation of smoking status and potential misclassification of infective respiratory complications), although the data analysis has limited the impact of this on our findings.^55^

Equally, sole reliance on patient identification through the trauma registry (TARN) risks missing patients who were coded incorrectly or missed from the database search could introduce selection bias. Furthermore, there is a small group of patients who attend the ED with isolated thoracic injuries without significant mechanisms of injury which are unlikely to be “trauma called” and therefore not identified by the trauma register.

This study has attempted to improve understanding of injury characteristics that predict increased risk for respiratory complications after significant blunt thoracic trauma. The analysis has also considered how these injury characteristics influence clinicians to use the various analgesic modes available in practice. Previous literature has demonstrated improved analgesia from the use of EA and PCA in this patient group. The link between adequate analgesia and reduced respiratory complications after an episode of blunt thoracic trauma has yet to be fully identified and the long-term benefit of analgesic mode has yet to be identified. For this to occur, further robust research must be undertaken. Until this occurs, the development of effective evidence-based local, national or international guidance remains difficult. For this reason, analgesic practice in trauma is unlikely to develop further without a stronger evidence base.

## References

[1] Willenberg L Curtis K Taylor C The variation of acute treatment costs of trauma in high-income countries. *BMC Health Serv Res* 2012; 12:267. 22909225 10.1186/1472-6963-12-267 PMC3523961

[2] Fingerhut LA Harrison J Holder Y Addressing the growing burden of trauma and injury in low- and middle-income countries. *Am J Public Health* 2005; 95:1089–1090. 15961751 10.2105/AJPH.2005.064469 PMC1449312

[3] D’Huyvetter C The trauma disease. *J Trauma Nurs* 2000; 7:5–12. 16422389 10.1097/00043860-200001000-00002

[4] Hofman K Primack A Keusch G Addressing the growing burden of trauma and injury in low- and middle-income countries. *Am J Public Health* 2005; 95:13–17. 15623852 10.2105/AJPH.2004.039354 PMC1449844

[5] World Health Organisation. World report on road traffic injury prevention. eds: Penden M. Scurfield R. Sleet D. Mohan D. Hyder A. Jarawan E 2004 World Health Organisation. Geneva, Switzerland

[6] Battle CE Hutchings H Evans PA Risk factors that predict mortality in patients with blunt chest wall trauma: a systematic review and meta-analysis. *Injury* 2012; 43:8–17. 21256488 10.1016/j.injury.2011.01.004

[7] Battle CE Hutchings H Evans PA Expert opinion of the risk factors for morbidity and mortality in blunt chest wall trauma: results of a national postal questionnaire survey of Emergency Departments in the United Kingdom. *Injury* 2013; 44:56–59. 22227106 10.1016/j.injury.2011.12.012

[8] Perna V Morera R Prognostic factors in chest traumas: a prospective study of 500 patients. *Cirugia Espanola* 2010; 87:165–170. 20074711 10.1016/j.ciresp.2009.11.020

[9] Natale C De Lesquen H Beranger F Blunt bronchial injuries: a challenging issue. *Injury* 2014; 45:3.23827395

[10] Bergeron E Lavoie A Clas D Elderly trauma patients with rib fractures are at greater risk of death and pneumonia. *J Trauma* 2003; 54:478–485. 12634526 10.1097/01.TA.0000037095.83469.4C

[11] Flagel BT Luchette FA Reed RL Half-a-dozen ribs: the breakpoint for mortality. *Surgery* 2005; 138:717–723.discussion 23–25. 16269301 10.1016/j.surg.2005.07.022

[12] Grossman MD Miller D Scaff DW When is an elder old? Effect of preexisting conditions on mortality in geriatric trauma. *J Trauma* 2002; 52:242–246. 11834982 10.1097/00005373-200202000-00007

[13] Holcomb JB McMullin NR Kozar RA Morbidity from rib fractures increases after age 45. *J Am Coll Surg* 2003; 196:549–555. 12691929 10.1016/S1072-7515(02)01894-X

[14] Blecher GE Mitra B Cameron PA Failed Emergency Department disposition to the ward of patients with thoracic injury. *Injury* 2008; 39:586–591. 18336817 10.1016/j.injury.2007.10.021

[15] Aguirre VJ Sinha P Zimmet A Phrenic nerve injury during cardiac surgery: mechanisms, management and prevention. *Heart Lung Circ* 2013; 22:895–902. 23948287 10.1016/j.hlc.2013.06.010

[16] Hua A Shah KH Does noninvasive ventilation have a role in chest trauma patients? *Ann Emerg Med* 2014; 64:82–83. 24126117 10.1016/j.annemergmed.2013.09.029

[17] Stewart RM Corneille MG Common complications following thoracic trauma: their prevention and treatment. *Semin Thorac Cardiovasc Surg* 2008; 20:69–71. 18420130 10.1053/j.semtcvs.2008.01.006

[18] DuBose JJ Putty B Teixeira PG The relationship between post-traumatic ventilator-associated pneumonia outcomes and American College of Surgeons trauma centre designation. *Injury* 2011; 42:40–43. 21595096 10.1016/j.injury.2009.08.026

[19] Unsworth A Curtis K Asha SE Treatments for blunt chest trauma and their impact on patient outcomes and health service delivery. *Scand J Trauma Resuscitation Emerg Med* 2015; 23:17. 10.1186/s13049-015-0091-5 PMC4322452 25887859

[20] Yeh DD Kutcher ME Knudson MM Epidural analgesia for blunt thoracic injury—which patients benefit most? *Injury* 2012; 43:1667–1671. 22704784 10.1016/j.injury.2012.05.022

[21] Asha SE Curtis KA Taylor C Patient-controlled analgesia compared with interval analgesic dosing for reducing complications in blunt thoracic trauma: a retrospective cohort study. *Emerg Med J* 2013; 30:1024–1028. 23221457 10.1136/emermed-2012-201980

[22] Bulger EM Edwards T Klotz P Epidural analgesia improves outcome after multiple rib fractures. *Surgery* 2004; 136:426–430. 15300210 10.1016/j.surg.2004.05.019

[23] Brasel KJ Guse CE Layde P Rib fractures: relationship with pneumonia and mortality. *Crit Care Med* 2006; 34:1642–1646. 16625122 10.1097/01.CCM.0000217926.40975.4B

[24] Carrier FM Turgeon AF Nicole PC Effect of epidural analgesia in patients with traumatic rib fractures: a systematic review and meta-analysis of randomized controlled trials. *Can J Anaesth* 2009; 56:230–242. 19247744 10.1007/s12630-009-9052-7

[25] Gage A Rivara F Wang J The effect of epidural placement in patients after blunt thoracic trauma. *J Trauma Acute Care Surg* 2014; 76:39–45.discussion -6. 24368355 10.1097/TA.0b013e3182ab1b08

[26] Marasco SF Davies AR Cooper J Prospective randomized controlled trial of operative rib fixation in traumatic flail chest. *J Am Coll Surg* 2013; 216:924–932. 23415550 10.1016/j.jamcollsurg.2012.12.024

[27] Chauny JM Emond M Plourde M Patients with rib fractures do not develop delayed pneumonia: a prospective, multicenter cohort study of minor thoracic injury. *Ann Emerg Med* 2012; 60:726–731. 22542306 10.1016/j.annemergmed.2012.03.020

[28] Barnea Y Kashtan H Skornick Y Isolated rib fractures in elderly patients: mortality and morbidity. *Can J Surg* 2002; 45:43–46. 11837920 PMC3692703

[29] Bulger EM Arneson MA Mock CN Rib fractures in the elderly. *J Trauma* 2000; 48:1040–1046.discussion 6–7. 10866248 10.1097/00005373-200006000-00007

[30] Perez MR Rodriguez RM Baumann BM Sternal fracture in the age of pan-scan. *Injury* 2015; 46:1324–1327. 25817167 10.1016/j.injury.2015.03.015

[31] Spencer Netto F Tien H Ng J Pulmonary emboli after blunt trauma: timing, clinical characteristics and natural history. *Injury* 2012; 43:1502–1506. 21722897 10.1016/j.injury.2010.12.028

[32] Vandenbroucke JP von Elm E Altman DG Strengthening the Reporting of Observational Studies in Epidemiology (STROBE): explanation and elaboration. *Ann Intern Med* 2007; 147:W163–W194. 17938389 10.7326/0003-4819-147-8-200710160-00010-w1

[33] Albaugh G Kann B Puc MM Age-adjusted outcomes in traumatic flail chest injuries in the elderly. *Am Surg* 2000; 66:978–981. 11261629

[34] Elmistekawy EM Hammad AA Isolated rib fractures in geriatric patients. *Ann Thorac Med* 2007; 2:166–168. 19727369 10.4103/1817-1737.36552 PMC2732099

[35] Harrington DT Phillips B Machan J Factors associated with survival following blunt chest trauma in older patients: results from a large regional trauma cooperative. *Arch Surg* 2010; 145:432–437. 20479340 10.1001/archsurg.2010.71

[36] Testerman GM Adverse outcomes in younger rib fracture patients. *South Med J* 2006; 99:335–339. 16634240 10.1097/01.smj.0000203815.29757.d3

[37] Liman ST Kuzucu A Tastepe AI Chest injury due to blunt trauma. *Eur J Cardiothorac Surg* 2003; 23:374–378. 12614809 10.1016/s1010-7940(02)00813-8

[38] Sirmali M Turut H Topcu S A comprehensive analysis of traumatic rib fractures: morbidity, mortality and management. *Eur J Cardiothorac Surg* 2003; 24:133–138. 12853057 10.1016/s1010-7940(03)00256-2

[39] Hoppe JA Kim H Heard K Association of emergency department opioid initiation with recurrent opioid use. *Ann Emerg Med* 2015; 65:493–499.e4. 25534654 10.1016/j.annemergmed.2014.11.015

[40] Wu CL Jani ND Perkins FM Thoracic epidural analgesia versus intravenous patient-controlled analgesia for the treatment of rib fracture pain after motor vehicle crash. *J Trauma* 1999; 47:564–567. 10498316 10.1097/00005373-199909000-00025

[41] Todd SR McNally MM Holcomb JB A multidisciplinary clinical pathway decreases rib fracture-associated infectious morbidity and mortality in high-risk trauma patients. *Am J Surg* 2006; 192:806–811. 17161098 10.1016/j.amjsurg.2006.08.048

[42] Trentzsch H Nienaber U Behnke M Female sex protects from organ failure and sepsis after major trauma haemorrhage. *Injury* 2014; 45 Suppl 3:S20–S28. 25284229 10.1016/j.injury.2014.08.013

[43] Harrison DA Welch CA Eddleston JM The epidemiology of severe sepsis in England, Wales and Northern Ireland, 1996 to 2004: secondary analysis of a high quality clinical database, the ICNARC Case Mix Programme Database. *Crit Care* 2006; 10:R42. 16542492 10.1186/cc4854 PMC1550902

[44] Orwelius L Lobo C Teixeira Pinto A Sepsis patients do not differ in health-related quality of life compared with other ICU patients. *Acta Anaesthesiol Scand* 2013; 57:1201–1205. 23895260 10.1111/aas.12164

[45] Eddleston JM White P Guthrie E Survival, morbidity, and quality of life after discharge from intensive care. *Crit Care Med* 2000; 28:2293–2299. 10921555 10.1097/00003246-200007000-00018

[46] Leone M Bregeon F Antonini F Long-term outcome in chest trauma. *Anesthesiology* 2008; 109:864–871. 18946299 10.1097/ALN.0b013e31818a4009

[47] Marasco S Lee G Summerhayes R Quality of life after major trauma with multiple rib fractures. *Injury* 2015; 46:61–65. 25069400 10.1016/j.injury.2014.06.014

[48] Dhillon TS Galante JM Salcedo ES Characteristics of chest wall injuries that predict postrecovery pulmonary symptoms: a secondary analysis of data from a randomized trial. *J Trauma Acute Care Surg* 2015; 79:179–187. 26218683 10.1097/TA.0000000000000718

[49] Honselmann KC Buthut F Heuwer B Long-term mortality and quality of life in intensive care patients treated for pneumonia and/or sepsis: predictors of mortality and quality of life in patients with sepsis/pneumonia. *J Crit Care* 2015; 30:721–726. 25818842 10.1016/j.jcrc.2015.03.009

[50] Rainer TH Yeung JH Cheung SK Assessment of quality of life and functional outcome in patients sustaining moderate and major trauma: a multicentre, prospective cohort study. *Injury* 2014; 45:902–909. 24314871 10.1016/j.injury.2013.11.006

[51] Green SM Krauss BS The numeric scoring of pain: this practice rates a zero out of ten. *Ann Emerg Med* 2015; doi: 10.1016/j.annemergmed.2015.06.002. 10.1016/j.annemergmed.2015.06.002 26116224

[52] Menger R Telford G Kim P Complications following thoracic trauma managed with tube thoracostomy. *Injury* 2012; 43:46–50. 21839442 10.1016/j.injury.2011.06.420

[53] Sethuraman KN Duong D Mehta S Complications of tube thoracostomy placement in the emergency department. *J Emerg Med* 2011; 40:14–20. 19097724 10.1016/j.jemermed.2008.06.033

[54] Corrales-Medina VF Alvarez KN Weissfeld LA Association between hospitalization for pneumonia and subsequent risk of cardiovascular disease. *JAMA* 2015; 313:264–274. 25602997 10.1001/jama.2014.18229 PMC4687729

[55] O’Reilly GM Cameron PA Jolley DJ Which patients have missing data? An analysis of missingness in a trauma registry. *Injury* 2012; 43:1917–1923. 22884761 10.1016/j.injury.2012.07.185

