# A Retrospective Observational Study Examining the Effect of Thoracic Epidural and Patient Controlled Analgesia on Short-term Outcomes in Blunt Thoracic Trauma Injuries: Erratum

**DOI:** 10.1097/01.md.0000481324.59838.57

**Published:** 2016-03-03

In the article “A Retrospective Observational Study Examining the Effect of Thoracic Epidural and Patient Controlled Analgesia on Short-term Outcomes in Blunt Thoracic Trauma Injuries”,^1^ which appeared in Volume 95, Issue 2 of *Medicine*, two minus signs were omitted from Table 4's Linear Regression 95% CI column. The article has since been corrected online.
